# Capacity-building efforts by the AFHSC-GEIS program

**DOI:** 10.1186/1471-2458-11-S2-S4

**Published:** 2011-03-04

**Authors:** Jose L Sanchez, Matthew C Johns, Ronald L Burke, Kelly G Vest, Mark M Fukuda, In-Kyu Yoon, Chanthap Lon, Miguel Quintana, David C Schnabel, Guillermo Pimentel, Moustafa Mansour, Steven Tobias, Joel M Montgomery, Gregory C Gray, Karen Saylors, Lucy M Ndip, Sheri Lewis, Patrick J Blair, Paul A Sjoberg, Robert A Kuschner, Kevin L Russell, David L Blazes, the AFHSC-GEIS Capacity Building Writing Group

**Affiliations:** 1 Armed Forces Health Surveillance Center, 503 Robert Grant Avenue, Silver Spring, MD 20910, USA; 2 Armed Forces Research Institute of Medical Sciences, 315/6 Rajavithi Road, Bangkok, Thailand 10400; 3 U.S. Army Public Health Command Region-South, Building 2472, Schofield Road, Fort Sam Houston, TX 78234, USA; 4 U.S. Army Medical Research Unit-Kenya, U.S. Embassy, Attn: MRU, United Nations Avenue, P.O. Box 606, Village Market 00621 Nairobi, Kenya; 5 Naval Medical Research Unit Number 3, Extension of Ramses Street, Adjacent to Abbassia Fever Hospital, Postal Code 11517, Cairo, Egypt; 6 Naval Medical Research Unit Number 2, Kompleks Pergudangan DEPKES R.I., JI. Percetakan Negara II No. 23, Jakarta, 10560, Indonesia; 7 Naval Medical Research Center Detachment-Peru, Centro Medico Naval “CMST,” Av. Venezuela CDRA 36, Callao 2, Lima, Peru; 8 Department of Environmental and Global Health, College of Public Health and Health Professions, University of Florida, Post Office Box 100188, Gainesville, FL 32610, USA; 9 Global Viral Forecasting Initiative, One Sutter Street, Suite 600, San Francisco, CA 94104, USA; 10 University of Buea, Department of Biochemistry and Microbiology, Faculty of Science, Post Office Box 63, Buea, South Western Province, Cameroon; 11 Johns Hopkins University Applied Physics Laboratory, 11100 Johns Hopkins Road, MP2-160, Laurel, MD 20723-6099, USA; 12 Naval Health Research Center, 140 Sylvester Road, San Diego, CA 92106, USA; 13 U.S. Air Force School of Aerospace Medicine, Public Health and Preventive Medicine Department, 2513 Kennedy Circle, Building 180, Brooks City-Base, TX 78235-5116, USA; 14 Walter Reed Army Institute of Research, Building 503, 503 Robert Grant Avenue, Silver Spring, MD 20910-7500, USA; 15 Kenyan Medical Research Institute, Mbagathi Post Office Box 54840, 00200, Nairobi, Kenya; 16 Landstuhl Regional Medical Center, CMR 402, Box 483, APO AE 09180, USA; 17 Makerere University Walter Reed Project, Plot 42, Nakasero Road, Post Office Box 16524, Kampala, Uganda; 18 Makerere University, Faculty of Veterinary Medicine & Medicine, Post Office Box 16524, Kampala, Uganda; 19 Navy Environmental and Preventive Medicine Unit Number 2, 1887 Powhatan Street, Norfolk, VA 23511-3394, USA; 20 PharmAccess Foundation, Skyway Building, Third Floor, Plot Number 149/32, Corner of Ohio Street/Sokoine Street, Post Office Box 635, Dar es Salaam, Tanzania; 21 Tanzania People’s Defence Forces, Defence Forces Headquarters Medical Services, Post Office Box 9203, Dar es Salaam, Tanzania; 22 U.S. Army Medical Department Activity & 65th Medical Brigade, Korea, Unit 15281, Box 769, APO AP 96205-5281; 23 U.S. Army Medical Research Institute of Infectious Diseases, Diagnostic Systems Division, 1425 Porter Street, Fort Detrick, MD 21702-5011, USA; 24 U.S. Army Public Health Command Region-Europe, Building 3810, CMR 402, Box 808, APO AE 09180; 25 U.S. Army Public Health Command Region-Pacific, Building 715, Camp Zama, Japan, Unit 45006, APO AP 96343-5006

## Abstract

Capacity-building initiatives related to public health are defined as developing laboratory infrastructure, strengthening host-country disease surveillance initiatives, transferring technical expertise and training personnel. These initiatives represented a major piece of the Armed Forces Health Surveillance Center, Division of Global Emerging Infections Surveillance and Response System (AFHSC-GEIS) contributions to worldwide emerging infectious disease (EID) surveillance and response. Capacity-building initiatives were undertaken with over 80 local and regional Ministries of Health, Agriculture and Defense, as well as other government entities and institutions worldwide. The efforts supported at least 52 national influenza centers and other country-specific influenza, regional and U.S.-based EID reference laboratories (44 civilian, eight military) in 46 countries worldwide. Equally important, reference testing, laboratory infrastructure and equipment support was provided to over 500 field sites in 74 countries worldwide from October 2008 to September 2009. These activities allowed countries to better meet the milestones of implementation of the 2005 International Health Regulations and complemented many initiatives undertaken by other U.S. government agencies, such as the U.S. Department of Health and Human Services, the U.S. Agency for International Development and the U.S. Department of State.

## Background

Capacity building, as it applies to health in this context, can be accomplished through strengthening health systems for delivery of medical care, pursuing medical research initiatives to answer important local or regional health questions, or supporting public health disease surveillance to prioritize which diseases are affecting relevant populations. Within this context, global public health capacity building can be defined as developing laboratory infrastructure, strengthening host-country disease surveillance initiatives, transferring technical expertise and training personnel. Disease surveillance is often the first step in improving public health because it attempts to quantify needs and allocate scarce assets in resource-limited settings, in addition to detecting potential outbreaks of disease.

Though not a new concept, capacity building has enjoyed renewed prominence as the world endeavors to meet requirements of International Health Regulations 2005 (IHR (2005)) [1]. Article 5 of the regulations requires that all countries be able to detect, assess, notify and report on public health issues of international significance and control any potential public health event of international concern by 2012. Some countries are capable now, but most are not and will not be compliant by the deadline unless a significant improvement in local capacity occurs. In general, for capacity building to be successful in the long term, efforts must not be undertaken quickly and need to be implemented through a concerted unified effort, achieving steady, sustainable and measurable progress over time, with the eventual goal being independence from the provider of the capability.

In 2007, the Government Accountability Office issued a report describing the global infectious disease capacity-building efforts of U.S. government (USG) entities [2]. At the time, three USG entities were identified as providing capacity building for emerging infectious diseases (EID), including the U.S. Centers for Disease Control and Prevention (CDC), the U.S. Agency for International Development and the Department of Defense’s Global Emerging Infections Surveillance and Response System (DoD-GEIS). Their efforts included laboratory-based disease surveillance, development and testing of diagnostics, and training such as Field Epidemiology Training Programs, the international version of the famed Epidemic Intelligence Service [3]. Currently, many other USG agencies are engaged in building disease surveillance capacity, including the U.S. Department of State, the Defense Threat Reduction Agency and the U.S. National Institutes of Health [4]. In addition, numerous state, non-state and non-governmental organizations, such as the Bill and Melinda Gates Foundation, the World Bank and Médecins sans Frontières, contribute substantially to capacity-building efforts around the world [5-7].

With the establishment of the Armed Forces Health Surveillance Center (AFHSC) in late 2008, the DoD-GEIS program was transitioned to a division and renamed “AFHSC-GEIS”; however, its mission of working to promote and facilitate national and international preparedness for EID was maintained. Strengthening of U.S. military and host-country disease surveillance and public health laboratory capacity represents a critical step for contributing to compliance with the IHR (2005) detection, reporting and response requirements. During 2009, capacity-building efforts were undertaken in a variety of formats, including enhancement of diagnostic capabilities, expansion of surveillance for militarily relevant infectious and tropical diseases, and deployment of electronic surveillance platforms. These efforts were coordinated with local host-country health officials and geographic Combatant Commands to ensure they addressed country and regional medical priorities as well as to ensure better surveillance and response to disease outbreaks and EID threats to U.S. forces abroad. These efforts focused on influenza and other respiratory diseases, malaria, dengue and other vector-borne illnesses, acute diarrheal diseases, antimalarial and antimicrobial resistance, sexually transmitted diseases, and bacterial wound infections.

## Accomplishments

### Laboratory infrastructure development

Capacity-building initiatives continued to represent a major component of AFHSC-GEIS contributions to worldwide EID surveillance and response activities. Inadequate laboratory capacity in developing countries has been termed the “Achilles’ heel” of global efforts to combat infectious diseases [8]. Thus, many AFHSC-GEIS sponsored activities in capacity building were directed at improving existing infrastructure by renovating current laboratory facilities, furnishing new scientific equipment, and provisioning new or enhanced diagnostic testing systems at overseas U.S. DoD facilities, as well as U.S.-based, DoD influenza reference laboratories, which serve as regional reference laboratories, and host-country laboratories.

Efforts were coordinated with over 80 local and regional Ministries of Health, Agriculture and Defense, as well as other government officials and institutions worldwide in 74 countries. A total of 52 National Influenza Centers (NICs) and other country-specific influenza and EID reference laboratories (44 civilian, eight military) were supported in 46 countries (Table 1). The efforts included support to laboratories in eight regions of the world. Sub-Saharan (east, central and west) Africa were the regions with the most major laboratory capacity-building efforts (in 14 countries), consistent with the identified needs of this region relative to the world, especially as it relates to influenza [9,10]. Among all infrastructure and capacity-building projects (Table 2), the majority supported primarily human health entities (in 67 countries); however, projects also supported animal health entities for zoonotic diseases in eight countries. Training efforts are mentioned, but are presented in detail elsewhere in this supplement [11].

**Table 1:** 2009 Major Laboratory Capacity-Building Initiatives by Geographic Region

Geographic Region	Major Laboratory Capacity Building Initiative	Countries Supported
Southeast Asia	NIC & military influenza lab equipment, reagent & training support; EID laboratory diagnostics & disease surveillance systems	Bhutan, Cambodia, Lao People’s Democratic Republic, Nepal, Singapore, Thailand
Far East	NIC & military influenza lab equipment & reagent support; EID lab proficiency & equipment support	Japan, Korea, Philippines
East & Central Africa	NIC & VHF lab equipment, reagent & training support; EID laboratory diagnostics	Cameroon, Kenya, Tanzania, Uganda
West Africa	NIC & MoH influenza lab equipment, reagent & training support; VHF lab diagnostics & military EID lab diagnostic testing capacity	Benin, Burkina Faso, Cote d’Ivoire, Ghana, Liberia, Mali, Niger, Nigeria, Sierra Leone, Togo
North Africa, Middle East & Southwest Asia	NIC lab equipment, reagent & training support	Afghanistan, Egypt, Iraq, Jordan, Kuwait, Oman, Pakistan, Sudan, Syria
Central Asia	EID & influenza lab equipment, reagent & training support	Azerbaijan, Georgia, Mongolia
Europe	Military & academic influenza lab equipment, reagent & training support	Poland, Romania
Central & South America	NIC & MoH influenza lab equipment, reagent & training support; leishmania military reference lab equipment, reagent & training support	Colombia, Ecuador, El Salvador, Guatemala, Honduras, Nicaragua, Panama, Paraguay, Peru

Acronyms: NIC, national influenza center; EID, emerging infectious diseases; VHF, viral hemorrhagic fever; MoH, Ministry of Health.

**Table 2:** 2009 Capacity-Building Initiatives by Major Regional AFHSC-GEIS Supported Partners and Type

Partner (see text)	Type of Infrastructure/Capacity Building*	Centers/Hospitals	Field Sites	Countries*
AFRIMS	Influenza & malaria/MDR labs (KH, PH); enteric & influenza lab upgrade (NP, TH); blood culture (NP); influenza testing (BT); influenza antiviral resistance (TH)	22	51	5
NAMRU-2	Malaria, FVBI, enteric, blood culture & AMR testing (KH); influenza & AFI testing (ID, KH, SG); surveillance data management (LA)	4	73	4
NAMRU-3	Influenza, blood culture & AMR testing (EG, JO); Influenza PCR/culture & antiviral resistance testing (32 countries); Joint Biological Agent Identification & Detection System (5 deployed US military sites-CENTCOM**); zoonotic disease & entomology (EG, DJ); AFI, blood/cerebrospinal spinal fluid culture & serology testing (AZ, GE); Leishmania PCR & culture (EG, LR); rotavirus testing (6 countries); cholera & other ADD testing (7 countries); FVBI testing (EG, DJ, AZ, GE)	37	42	34
NMRCD-Peru	Influenza PCR/culture & antiviral resistance testing support (10 countries); AFI & viral culture & serology testing (PE, BO, EC, PY); Leishmania PCR, MDR, urine/vaginal PCR-STIs, Rickettsial PCR & culture (PE); enteric culture, PCR & AMR testing (PE, EC, PY); Alerta electronic disease surveillance system (PE, PA, EC)	23	102	11
USAMRU-Kenya	Malaria/MDR, microscopy & PCR, rotavirus, cholera & other ADD testing, arboviral/VHF PCR & culture, AFIs, blood culture & serology testing, STIs culture (KE); influenza PCR, culture &genotyping (KE, UG, CM); influenza, AFI, FVBI, cholera & other ADDs (KE, TZ, NG)	7	69	5
PHCR-South	Influenza PCR, culture & indirect immunofluorescence assay (US, HN, SV, NI, GT, PA); malaria, Leishmania, & dengue PCR testing (HN)	4	7	6
Univ Iowa CEID	Respiratory & other zoonotic respiratory EID testing & epidemiology (US, TH, KH, NG, RO, MN)	6	~30	6
JHU/APL	Influenza military treatment facilities (PIPM) modeling (US); SMS text & ESSENCE Desktop edition system (PH); Open source Interactive Voice Recognition software surveillance (PE); OpenESSENCE website software surveillance (US, PE); SMS text (PH)	1	~125	3

Acronyms: MDR, multidrug resistance; FVBI, febrile & vector-borne illnesses; AMR, antimicrobial resistance; AFI, acute febrile illnesses (such as dengue, leptospirosis and zoonotic infections); PCR, polymerase chain reaction; ADD, acute diarrheal diseases (such as traveler’s diarrhea, campylobacter, shigellosis, salmonellosis); STIs, sexually transmitted infections, including *Neisseria gonorrhea*; EID, emerging infectious diseases; PIPM, Pandemic Influenza Prevention Modeling; SMS, Short Message Service; ESSENCE, Electronic Syndromic Surveillance for Early Notification of Community-based Epidemics.

* Country names are displayed in parenthesis using the International Organization for Standardization (ISO 3166) two-character code (URL: http://www.commondatahub.com/live/geography/country/iso_3166_country_codes?gclid=CPSnst2e5KQCFQqP5god3xzd8A); Countries column represents the number where activities have been implemented; U.S. military deployment sites (such as Iraq, Afghanistan) or U.S. Department of State embassies do not contribute to separate country counts, since they represent overseas locations where U.S. forces and/or civilians are deployed or stationed.

** CENTCOM, U.S. Central Command (forward U.S. troop deployment sites).

**Figure 1:**
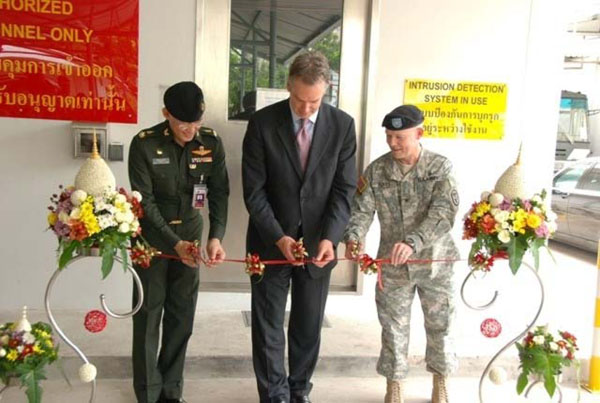
**AFRIMS BSL-3 Laboratory Commissioning**. On September 16, 2009 (from left to right), Major General Krisada Duangurai, director general of AFRIMS; U.S. Ambassador Eric John, together with Colonel James Boles, commander of AFRIMS, officiated the ribbon-cutting ceremony for the AFRIMS BSL-3 laboratory. This facility significantly contributes to the country’s capacity to conduct research and investigate outbreaks caused by agents, such as avian influenza, chikungunya virus and other endemic diseases throughout Southeast Asia.

One of the most notable AFHSC-GEIS accomplishments in fiscal 2009 was the establishment of two new biosafety level-3 (BSL-3) laboratory suites within DoD reference laboratories. The Armed Forces Research Institute of Medical Sciences (AFRIMS) in Bangkok, Thailand, completed the first laboratory, which the United States certified and commissioned on July 8, 2009. The suite was officially inaugurated September 16, 2009 and began immediately supporting work in avian and pandemic influenza monitoring, including culture and molecular sequencing capability (Figure 1). This BSL-3 laboratory constitutes the first DoD-certified laboratory of its kind in the region and provides the World Health Organization (WHO), Thailand and other countries in Southeast Asia with a much-needed high-containment capability to conduct research and assist with outbreaks involving select human and animal bacterial and viral strains.

**Figure 2:**
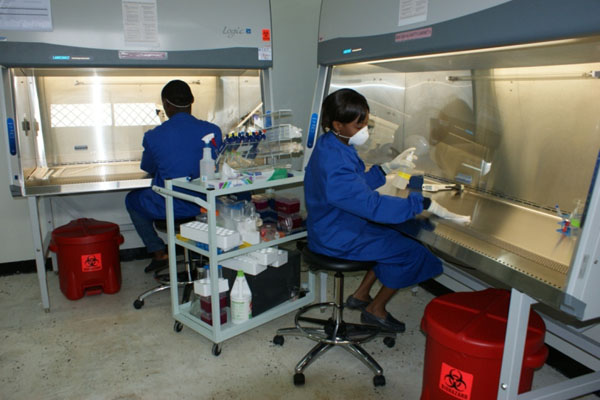
**Influenza Surveillance Capacity-Building Initiative with Global Viral Forecasting Initiative and University of Buea, Cameroon.** Two biosafety level-2 laboratories were renovated at the Cameroon Army Military Health Research Center in Yaoundé and at the University of Buea, in cooperation with the Cameroon government and military. These laboratories have the capacity to isolate and characterize human and animal influenza viruses, as well as other EID pathogens of unknown origin.

The Naval Health Research Center (NHRC) opened a second BSL-3 (agriculture-enhanced) laboratory suite in late 2009. The facility allows work with zoonotic influenza strains submitted by AFHSC-GEIS partners around the world, including development of new virus neutralization testing capabilities against H5N1 and other highly pathogenic avian influenza strains. Additionally, two BSL-2 laboratories were also established at the Cameroon Army Military Health Research Center, supported by Global Viral Forecasting Initiative in Yaoundé and at the University of Buea (Figure 2). Both facilities will greatly improve the ability to conduct influenza and EID diagnostic work, as well as potentially advanced pathogen discovery work in hard-to-reach locations in Africa.

Efforts were also undertaken to improve laboratory capability for global influenza surveillance and diagnosis, especially regarding the novel A/H1N1 influenza pandemic. To this end, AFRIMS established viral/bacterial pathogen culture and molecular diagnostic capability in their Nepal detachment to support the National Public Health Laboratory and also established real-time reverse transcriptase polymerase chain reaction (rRT-PCR) diagnostic capacity for influenza at a main tertiary-care hospital of the Department of Health within the Visayas region of the Philippines.

Developing influenza diagnostic capabilities at other NICs was also supported by the U.S. Naval Medical Research Unit No. 3 (NAMRU-3) in Afghanistan, Iraq and Jordan; by the U.S. Naval Medical Research Center Detachment in Peru (NMRCD-Peru) in the countries of Colombia, Ecuador, Paraguay and Venezuela; and in Kenya, by the U.S. Army Medical Research Unit-Kenya. Finally, in conjunction with the CDC’s Central America and Panama center, the U.S. Army Public Health Command Region-South (PHCR-South) provided laboratory technical assistance, reagents and supplies to the Ministries of Health (MoHs) in El Salvador, Guatemala, Honduras, Nicaragua and Panama, resulting in the certification of the Guatemalan NIC and the testing of over 5,000 specimens for novel A/H1N1.

In collaboration with the Peruvian Navy, NMRCD-Peru has built a robust shipboard disease surveillance infrastructure with detection capability modeled very closely on the NHRC shipboard surveillance system. The early detection aspect of this system involves equipping participating ships with real-time PCR diagnostic capability for emerging infectious diseases, such as influenza or adenovirus. Short-term storage of samples allows for more in-depth, follow-up testing at the laboratory in Lima or at other collaborating regional laboratories. Since 2007, this system has successfully identified and responded to numerous outbreaks of respiratory, gastrointestinal and sexually transmitted infections among active-duty Peruvian personnel aboard ships [12]. More recently, this capability was instrumental in identifying and responding to a large outbreak of novel A/H1N1 on board a large deck ship in the Pacific [13].

This investment in laboratory infrastructure development has directly impacted the number of outbreak investigations that the AFHSC-GEIS network has been able to support. The capacity-building efforts contributed to outbreak responses in 76 instances in 53 countries, representing every major populated region of the world, including support for the confirmation of the first cases of novel A/H1N1 in 14 countries (United States, Bhutan, Cambodia, Colombia, Djibouti, Ecuador, Egypt, Kenya, Kuwait, Lao People’s Democratic Republic (PDR), Lebanon, Nepal, Peru and the Republic of the Seychelles) [12]. The laboratory infrastructure allows for acute response capability and the ability to monitor ongoing epidemics or shifting EID patterns, such as the identification and continued monitoring of artemisinin-resistant malaria in Southeast Asia by partners from AFRIMS [14] and at the U.S. Naval Medical Research Unit No. 2 (NAMRU-2) or the search for genetic mutations within influenza viruses that may indicate resistance to antiviral medications.

## Training

It is important to recognize that capacity building not only involves renovating laboratories and providing diagnostic equipment and supplies, but most important, building human capacity. Through training public health and laboratory personnel, the physical infrastructure could be properly leveraged for optimal support of IHR (2005) compliance. During 2009, AFHSC-GEIS supported 18 partner organizations that conducted 123 training initiatives in 40 countries involving at least 3,130 people, including many host-country personnel, in direct support of assisting with compliance with IHR (2005). Significant expansion of training activities was attained in the areas of pandemic preparedness, outbreak investigation and response, EID surveillance, and pathogen diagnostic techniques.

By engaging local health and other government officials and civilian institutions in training endeavors, the U.S. military’s role as a key stakeholder in global public health has improved; and many opportunities for EID-related surveillance, research and capacity-building initiatives have been leveraged to provide a platform for public health training, described elsewhere in this supplement [11].

## Electronic surveillance initiatives

Electronic disease surveillance, another important component of a comprehensive global public health disease prevention and control strategy, contributes significantly to capacity building and support for IHR (2005) compliance in partner countries. Using electronic methods for data collection and analysis has the potential to improve the accuracy and timeliness of outbreak detection, as well as to provide situational awareness during, or in the aftermath of, an outbreak or pandemic. The AFHSC-GEIS network has supported numerous initiatives in electronic disease surveillance during the past several years, in partnership with several DoD overseas laboratories, host-country Ministries of Health and Defense and our technical partner, the Johns Hopkins University Applied Physics Laboratory (JHU/APL).

AFHSC-GEIS has relied on the extensive experience that JHU/APL acquired in the design and implementation of the Electronic Syndromic Surveillance for Early Notification of Community-based Epidemics (ESSENCE) system [15]. This electronic disease surveillance system, used worldwide at all DoD military treatment facilities (MTFs), the U.S. Veterans Health Administration system and at least 12 states in the United States, served as a model for a toolkit approach to deploying electronic surveillance within the AFHSC-GEIS network. Tools have been created to enable data collection from the most sophisticated data sources to remote settings where data have traditionally been difficult, if not impossible, to collect. These tools have far-reaching applicability in any resource-limited setting, whether overseas or after a disaster in the United States. The following describes some of the efforts that have focused on adapting electronic or syndromic surveillance techniques to resource-limited settings.

Two electronic surveillance efforts were developed at AFRIMS in Southeast Asia and optimized in 2009, including a project with the Royal Thai Army (RTA) in remote border areas, as well as a pilot short message service (SMS)-based project in the Philippines, part of a joint effort with JHU/APL and the Cebu City Health Office (CHO). The Thai Unit-Based Surveillance (UBS) project commenced in 2001 and originally covered areas along the Thai-Cambodia border where the Thai MoH did not have disease surveillance capabilities. The project, developed by the RTA with support from AFRIMS and AFHSC-GEIS, reports diseases in both military and local civilian populations by faxing reports or by voice via military radio. In 2009, the Thai-Myanmar border area was added and an additional 497 personnel were trained. Version 2.0 of the UBS simplified data collection from 216 symptoms and categorization into 12 syndromes that are consistent with the Thai MoH’s reporting requirements. This updated system added questions about poultry exposure, leptospirosis, novel A/H1N1 infection and chickungunya virus infection. Although no major outbreaks of disease were detected by this system in 2009, it continued to provide situational awareness for the RTA and Thai MoH.

Dengue fever poses a significant health threat in the Philippines. Current hospital-based surveillance is highly valid, but poorly suited for rapid identification of dengue ”hot spots” because of delays associated with laboratory confirmation. To capture this important data for the purposes of surveillance, a more rapid, but less specific surveillance method was implemented and compared to the standard sentinel surveillance system. This pilot study implements and evaluates a simple dengue surveillance protocol using SMS text messages to send daily, person-based dengue surveillance data from local Barangay Health Centers (BHCs) to the city health office (CHO) in Cebu City. The pilot activity was originally established in five clinics as of March 2009, but was soon instituted in all BHCs in the city. Beginning July 1, 2009, all BHCs have been identifying all patients reporting to clinic with fever. Each day, BHC personnel send this information to the CHO, creating a text message for each patient with fever. The SMS message contains the date and clinic name, as well as the patient’s name, age, gender and symptoms. The message is transferred into a Microsoft Access© database, cleaned, and starting July 2010, reviewed in the ESSENCE Desktop Edition application to identify statistically significant increases in reported fever cases.

Meanwhile, NAMRU-2 continued to support the optimization of the Early Warning Outbreak Recognition System (EWORS) at 11 reference and provincial hospitals in the Lao PDR allowing local MoH officials to monitor the impact and burden of tropical and infectious diseases in the country in real time. The CDC currently funds most of the operating budget for EWORS in Lao PDR. The system, jointly developed by the Indonesian MoH and NAMRU-2 with AFHSC-GEIS funding, is also being used in Indonesia as the national reporting system. EWORS has additionally been used in Cambodia, Peru and Vietnam, although it is no longer in use in these countries because local health authorities favored other surveillance systems.

In South America, NMRCD-Peru supported major efforts in electronic disease surveillance, including continuation and optimization of Alerta, a public-private initiative that has revolutionized surveillance for the Peruvian military during the past seven years. The Alerta system has seen recent expansion to all branches of the Peruvian military, as well as adoption by the MoH of one other country in the region—Panama. This system identified 17 outbreaks during 2009, including influenza, dengue, mumps, malaria, hepatitis A and respiratory disease.

Finally, in collaboration with the JHU/APL group, NMRCD-Peru worked to develop an electronic syndromic surveillance system based on open-source software for use in resource-limited environments. As a result, the system can be sustained without continued major investments or software licensing fees. This effort involved the development of interactive voice response reporting, as well as building a web-based infrastructure and database on an open-source version of the ESSENCE system (OpenESSENCE) in use in the United States. Additionally, NMRCD-Peru supported the systematic evaluation of these electronic surveillance systems and research on ways to improve reporting via electronic systems [16].

These electronic surveillance initiatives constitute a vibrant portfolio that capitalizes on the expertise of the JHU/APL group and numerous AFHSC-GEIS partners at overseas laboratories and within host-country Ministries of Health and Defense. Many of the lessons learned, challenges, successes and failures have been shared within this network of collaborators, and a harmonized strategy is emerging to develop and deploy an electronic disease surveillance system that is modular and responsive to various needs found in developing settings. This approach should assist many countries in complying with IHR (2005) by the 2012 deadline.

## Provision of technical expertise/reference laboratory support

In addition to supporting laboratory infrastructure development and new surveillance initiatives, AFHSC-GEIS provided technical expertise in support of capacity-building efforts. In 2009, one of the largest such efforts was the network’s global response to the novel A/H1N1 influenza pandemic. For example, NAMRU-3 provided training on laboratory techniques for 73 scientists and technical personnel from 32 countries in western and northern Africa, the Middle East, and central Asia, as well as equipment and reagent support to established NICs in Egypt, Kuwait, Oman, Pakistan, Sudan and Syria. Support for further viral characterization by genetic sequencing and antiviral resistance testing was also performed at NAMRU-3, with reference testing support by the CDC in Atlanta. This virology diagnostic-testing capacity building of national reference laboratories constituted an essential step in establishing the capability for H5N1 and novel A/H1N1 detection and rapid response, and resulted in a better understanding of the epidemiologic patterns of respiratory viruses circulating in the region. It also represented the first step toward NIC accreditation and collaboration with the WHO Global Influenza Surveillance Network in support of influenza vaccine development. By linking countries in regional and sub-regional networks and by fostering participation in WHO missions to assess laboratory testing capacity needs, NAMRU-3 played a direct role in promoting IHR (2005) compliance.

Working closely with U.S. Central Command and U.S. Africa Command, NAMRU-3 and the U.S. Navy Environmental and Preventive Medicine Unit No. 2 (NEPMU-2) provided focused laboratory assessment, training, emergency supplies and quality assurance support to five military, far-forward deployed, influenza testing laboratories in Southwest Asia and assisted with the deployment of the Joint Biological Agent Identification and Detection System (JBAIDS) platform for confirmation of novel A/H1N1 cases in-theater. This capability subsequently proved critical when Expeditionary Medical Forces in Kuwait and Djibouti were able to identify and respond to novel A/H1N1 and seasonal influenza outbreaks, respectively.

Network expertise and competence were important in supporting global influenza testing efforts. For instance, the AFRIMS-supported laboratory in the Philippines was designated by the Philippine NIC as the only other facility authorized to conduct novel A/H1N1 testing, in support of central and southern regions of the country (specifically, Mindanao and Visayas).

## Military-to-military (mil-mil) partnerships

Growing collaborative military-military partnerships and surveillance exchanges among global network partners and foreign military counterparts continued to be an area of high interest and priority for AFHSC-GEIS. The network currently supports active military partnerships in 14 countries. These partnerships resulted in a number of collaborative response activities that supported foreign military partners, multinational peacekeepers and observers in joint exercises and missions.

The late spring and summer outbreaks of novel A/H1N1 in military treatment facilities throughout Europe resulted in collaboration between Landstuhl Regional Medical Center and PHCR-Europe and the German Military Reference Laboratory. The long-standing relationship between the U.S. European Command and the German Army’s Public Health Service helped assist in disseminating confirmed results through weekly surveillance reports sent to military clinicians, hospital commanders, and other public health officials within the U.S. military and the local German public health infrastructure. This arrangement greatly aided the U.S. European Command’s ability to conduct surveillance for novel A/H1N1 within the European military community and assisted German government officials in monitoring the level of disease within their country.

Efforts have been established to collaborate on more expansive and cross-cutting surveillance systems with military partners in Poland and Singapore. These efforts include a wide spectrum of surveillance from electronic early detection systems and routine laboratory-based sentinel surveillance to robust pathogen discovery initiatives and focused public health research endeavors. Collaboratively, these efforts have developed significantly during the past year and have helped serve as a model for other AFHSC-GEIS partners to engage their regional foreign military counterparts. These mil-mil partnerships with allied countries allow for open collaboration, capacity building and transparent dialogue between partner countries, and thus have the potential to develop a meaningful framework to better understand disease dynamics among military populations in different parts of the world. To further foster opportunities for these mil-mil partnerships, AFHSC-GEIS is working with the International Congress on Military Medicine and the WHO by facilitating educational opportunities with regard to IHR (2005) and creation of a portfolio of robust epidemiological tools and training that member countries can access as needed [17].

## Future directions and challenges

Significant progress was attained in expansion of worldwide EID surveillance and response initiatives in fiscal 2009 through the capacity-building efforts of the AFHSC-GEIS network described above. At this juncture, however, it is necessary to achieve realistic goals in terms of maturation, standardization and unification of the division’s global surveillance efforts. This can best be accomplished by pursuing the following strategic goals: 1) adopting objective metrics of evaluation, such as timeliness of disease detection and reporting to higher levels, proportion of sites submitting timely weekly or monthly reports, proportion of investigated outbreaks with confirmed laboratory results, and proportion of confirmed outbreaks with nationally recommended public health response [18]; 2) ensuring future standardization of genetic and molecular-based testing platforms (e.g., PCR-based assays) across the network of partners; 3) establishing electronic sequence data repositories for more effective information sharing with the CDC, WHO and local regional health authorities (especially for influenza and other respiratory pathogens); 4) continuing emphasis on collaborative work with host-country partners to empower them to reach IHR (2005) capacity-building milestones by 2012; and, 5) achieving standardized reporting schemes for all AFHSC-GEIS partners in the areas of influenza, enteric diseases, febrile and vector-borne illnesses, sexually transmitted infections, and antimicrobial resistance monitoring. In this manner, the AFHSC-GEIS network will continue to contribute to the global efforts in disease control and prevention through the DoD’s laboratory-based surveillance and by enhancing harmonization of efforts with other key USG stakeholders, such as the U.S. Department of Health and Human Services, the U.S. Agency for International Development and the U.S. Department of State.

Many challenges exist to building capacity for public health in resource-limited settings, including achieving sustainability of efforts after support is withdrawn, containing the departure of highly-trained, capable scientists after training, and minimizing the duplication of efforts among multiple sponsor agencies within the USG and with other organizations. Data sovereignty and data sharing are also key issues that require transparency on the part of both the sponsor and recipient in order to optimally conduct disease surveillance that satisfies the spirit of IHR (2005). Solutions to many of these challenges are sometimes difficult and frequently require continuous re-evaluation of best of practice solutions for individual settings.

Through the development of active, mutually supportive relationships with local health officials and the establishment of important protocol-driven clinical and laboratory surveillance projects, AFHSC-GEIS supported scientists have become relevant stakeholders within host-country public health communities and are able to continue to work in the critical development of surveillance, laboratory and communications infrastructure within partner countries. In addition to the IHR (2005), the AFHSC-GEIS global network recognizes the recently released National Strategy for Countering Biological Threats (PPD-2) as another guiding framework for alignment of our program with the larger USG initiatives [19], keeping the maintenance of the U.S. military’s health (known as “Force Health Protection”) as our unique niche in the setting of improving global public health. Meaningful public health initiatives taking place in any one of the partner countries within the AFHSC-GEIS global network must aim for incremental, albeit sustainable, development of capacity on behalf of their partner host countries and do so in line with the specific PPD-2 objectives and IHR (2005) competencies. In this manner, small improvements in capacity, improved testing abilities, and ultimately, compliance with reporting will lead to benefits for the health of U.S. servicemembers and for the health of the world.

## Competing interests

To the best knowledge of the authors, there are no competing interests.
